# 1878. Short message service (SMS) alert and reschedule an appointment to shorten physician delay of TB treatment: A quasi-experimental study

**DOI:** 10.1093/ofid/ofad500.1706

**Published:** 2023-11-27

**Authors:** Nasikarn Angkasekwinai, Theerajet Guayboon, Popchai Ngamskulrungroj

**Affiliations:** Faculty of Medicine Siriraj Hospital, Mahidol University, Bangkok, Krung Thep, Thailand; Siriraj Hospital, Nonthaburi, Nonthaburi, Thailand; Siriraj Hospital, Nonthaburi, Nonthaburi, Thailand

## Abstract

**Background:**

Tuberculosis (TB) continues to pose a major health threat during the ongoing coronavirus diseases 2019 (COVID-19) pandemic in Thailand. Delays of TB treatment either due to patients or healthcare system resulted in an unfavorable outcome. There is limited study using information technology to diminish physician delay of TB treatment.

**Methods:**

This was a quasi-experimental study conducted from June 2019 to February 2022 at Siriraj Hospital to assess the impact of using the short message service (SMS) alerts and rescheduling an appointment to shorten the physician delay in starting TB treatment. Patients were eligible if they were ≥18 years and having a positive TB test results by either one of the following: acid-fast bacilli (AFB) smear, polymerase chain reaction (PCR) for TB, or mycobacterial culture reporting *M. tuberculosis* complex. The primary outcome was the duration from reporting the first positive TB test to the treatment initiation.

**Figure:**
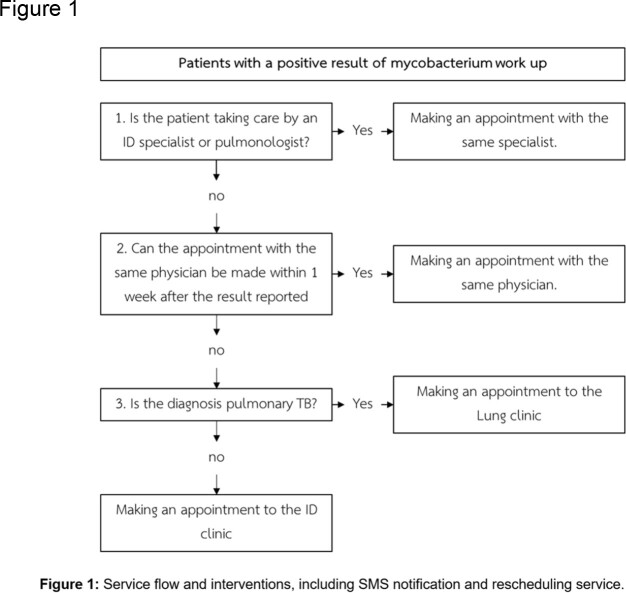

Service flow and interventions, including SMS notification and rescheduling service.

Figure 2

**Figure:**
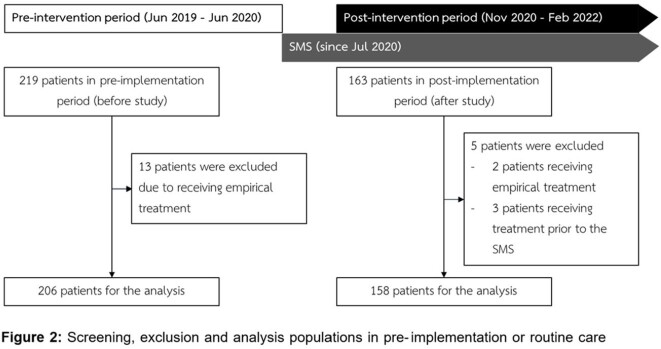

Screening, exclusion and analysis populations in pre-implementation or routine care and post-implementation of SMS service.

**Results:**

Among 364 patients, 206 patients and 158 patients were included in the no SMS and the SMS groups, respectively. The SMS group had a significantly higher proportion of being treated in the OPD setting (94.3% vs 67%, p< 0.001), treated by staff (42% vs. 23.5%, p< 0.001), and treated by pulmonologists (54.4% vs. 36.2%, p=0.004) than the no SMS group. Patients in the SMS group had a longer median time from the first visit to treatment (29 days [14.0, 57.8] vs.12 days [5.0, 47.5], p< 0.001), specimen collection to treatment (15.5 days [7.0, 31.8] vs. 6.5 days [2.0, 22.8], p< 0.001), and reporting results to treatment (9 days [5.0, 16.0] vs. 3.5 days [1.0, 12.0], p< 0.001) than the no SMS group. However, among TB patients being treated in the OPD setting and having a culture as the first positive result, those in the SMS group had a significantly shorter median time from specimen collection to treatment than the no SMS group (36 days [29.0, 51.0] vs. 60 days [39.0, 101.0], p=0.018). The median time from reporting the results to treatment was also shorter in the SMS group (9 days [6.0, 30.0] vs. 25 days [6.3, 81.0], p=0.172) but there was no statistically significant difference.

**Figure:**
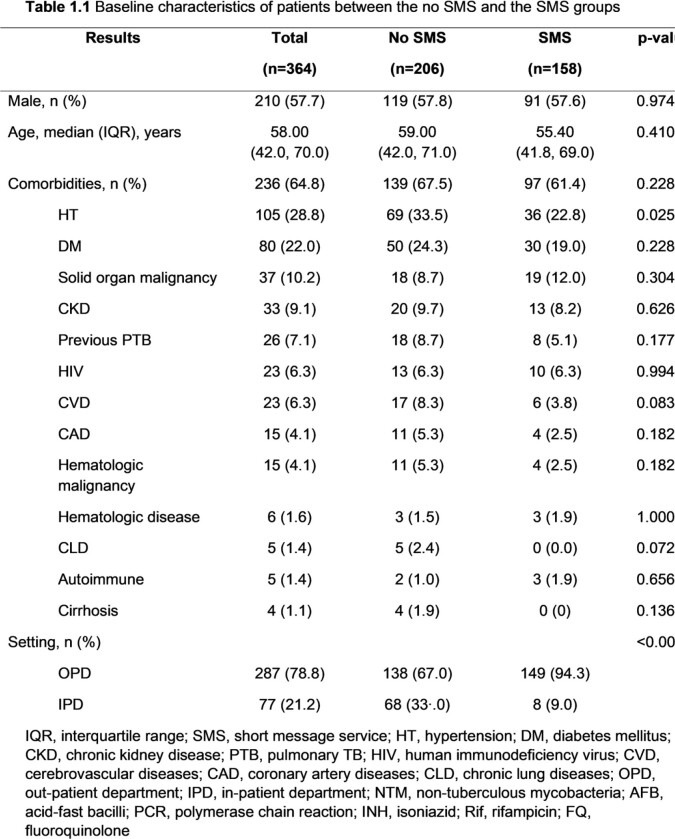

**Figure:**
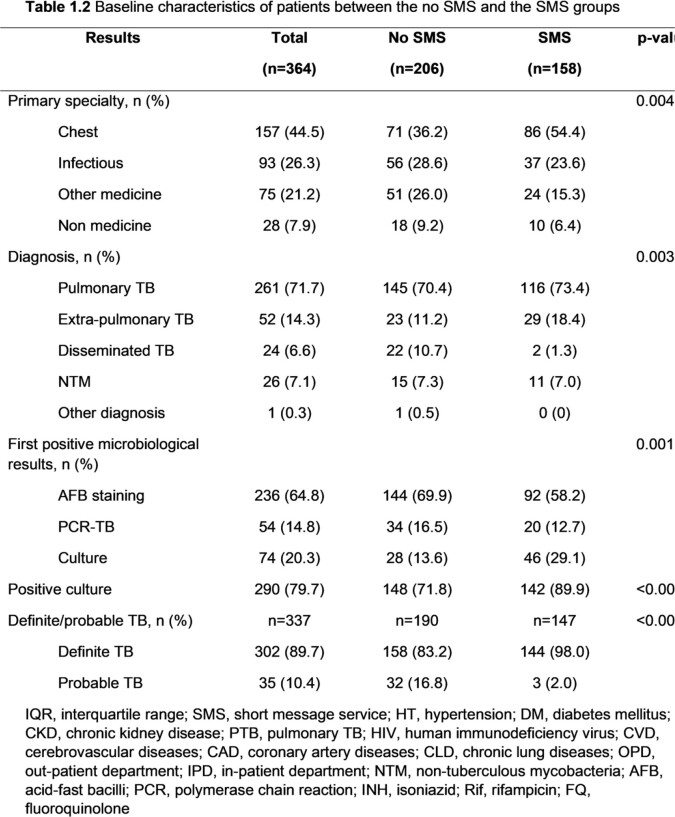

**Figure:**
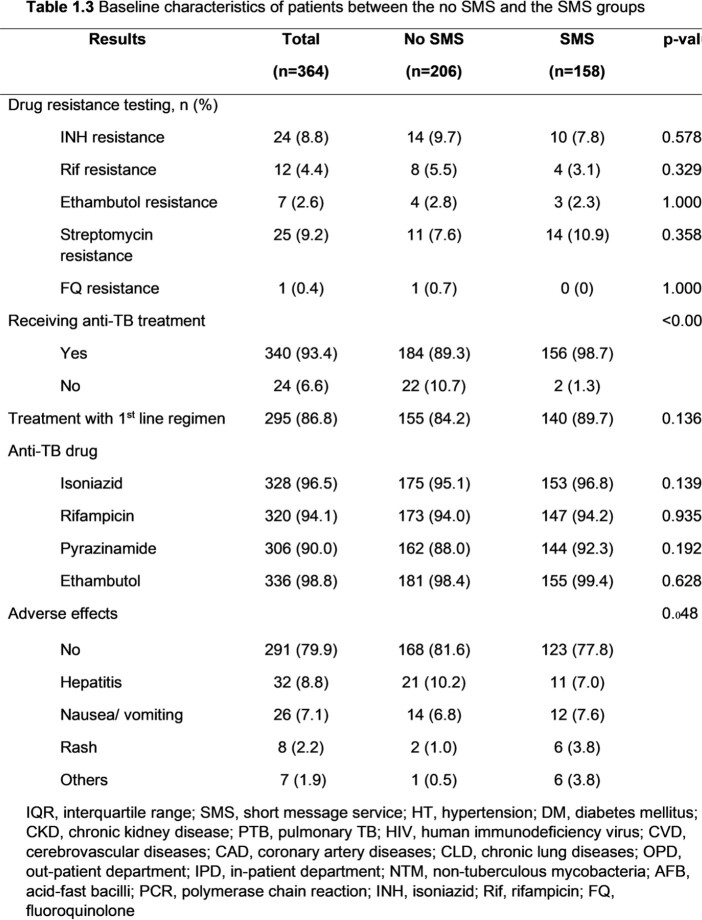

**Conclusion:**

SMS notification could reduce healthcare system delays in the initiation of TB treatment among TB patients who have positive mycobacterial cultures, but negative AFB smear and PCR-TB.

**Figure:**
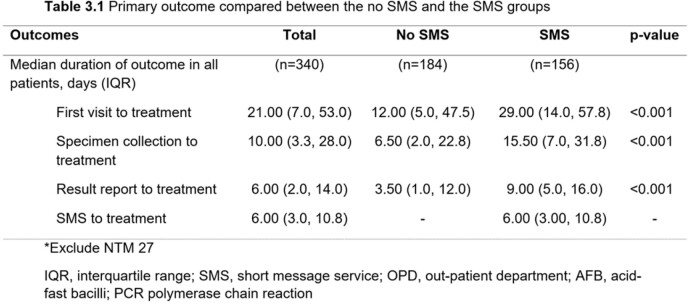

**Figure:**
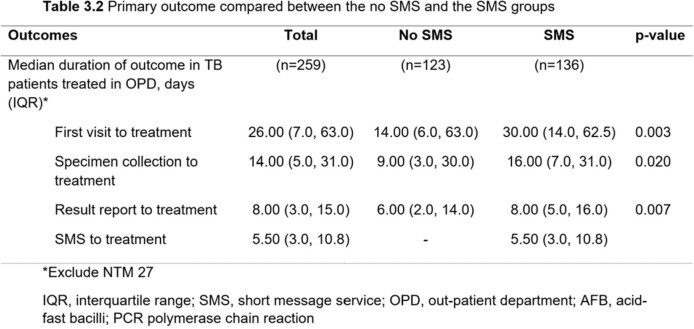

**Figure:**
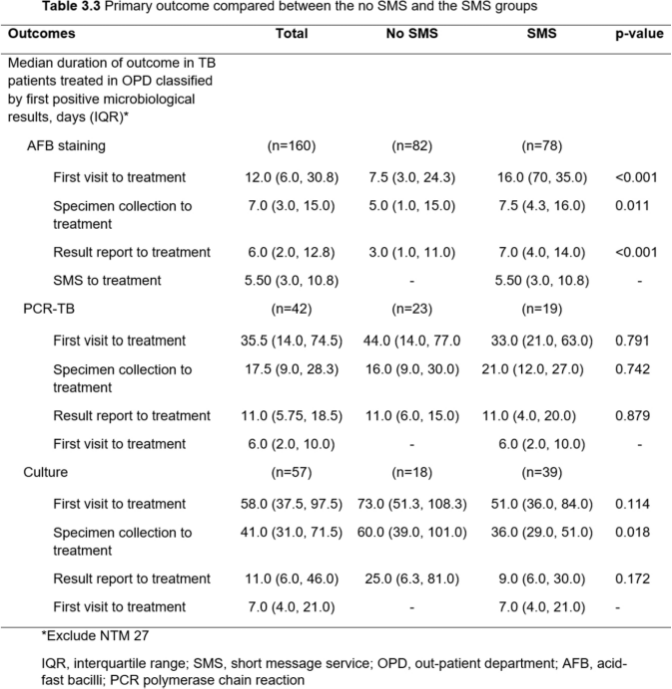

**Disclosures:**

**All Authors**: No reported disclosures

